# Effects of Carboxyl or Amino Group Modified InP/ZnS Nanoparticles Toward Simulated Lung Surfactant Membrane

**DOI:** 10.3389/fbioe.2021.714922

**Published:** 2021-08-19

**Authors:** Juan Wang, Shun Feng, Jie Liu, Rui-Lin Liu

**Affiliations:** ^1^ Shaanxi Engineering Research Center of Controllable Neutron Source, School of Science, Xijing University, Xi’an, China; ^2^ School of Pharmacy, Xuzhou Medical University, Xuzhou, China

**Keywords:** pulmonary surfactant monolayer, elastic modulus, Brewster angle microscope, InP/ZnS QDs, carboxyl and amino groups

## Abstract

Quantum dots (QDs) as a promising optical probe have been widely used for *in vivo* biomedical imaging; especially enormous efforts recently have focused on the potential toxicity of QDs to the human body. The toxicological effects of the representative InP/ZnS QDs as a cadmium-free emitter are still in the early stage and have not been fully unveiled. In this study, the DPPC/DPPG mixed monolayer was used to simulate the lung surfactant monolayer. The InP/ZnS-COOH QDs and InP/ZnS-NH_2_ QDs were introduced to simulate the lung surfactant membrane’s environment in the presence of InP/ZnS QDs. The effects of InP/ZnS QDs on the surface behavior, elastic modulus, and stability of DPPC/DPPG mixed monolayer were explored by the surface pressure-mean molecular area isotherms and surface pressure-time curves. The images observed by Brewster angle microscope and atomic force microscope showed that the InP/ZnS QDs affected the morphology of the monolayer. The results further demonstrated that the InP/ZnS QDs coated with different surface groups can obviously adjust the mean molecular area, elastic modulus, stability, and microstructure of DPPC/DPPG mixed monolayer. Overall, this work provided useful information for in-depth understanding of the effects of the −COOH or −NH_2_ group coated InP/ZnS QDs on the surface of lung surfactant membrane, which will help scientists to further study the physiological toxicity of InP/ZnS QDs to lung health.

## Introduction

Quantum dots (QDs) are one kind of the most classical quasi-zero dimensional and semiconductor nanocrystals, which have special optical properties: fluorescence, phosphorescence, and electro-chemiluminescence (Bruchez et al., 1998; Namdari et al., 2017; Pohanka, 2017). They have been used in various areas of human life (Pelley et al., 2009; Matea et al., 2017; Zhao et al., 2018), such as electronic and quantum computing applications, *in vitro* diagnostics, and *in vivo* biomedical imaging (Hong et al., 2012; Wang and Yan, 2013; Chen et al., 2014; Zhang et al., 2018). Typically, the cadmium-containing quantum dots as the foremost probe were rapidly developed, but the released cadmium ions can cause dramatic damage to cells and organs (Li et al., 2009; Wang et al., 2016). The InP/ZnS QDs as a core/shell type cadmium-free nanoparticle can effectively replace cadmium-containing quantum dots because the former has relatively lower toxicity (Chibli et al., 2011). In fact, the potential toxicity of the InP/ZnS QDs has not been fully explored since it is still in the early stage.

Soenen et al. (2014) has investigated the toxicity of InP/ZnS QDs on the cells *in vitro* using three different cell types: primary human umbilical vein endothelial cells (HUVEC), murine neural progenitor cells (C17.2), and rat pheochromocytoma cells (PC12). They found that the InP/ZnS QDs can be ingested efficiently by the three cell types and the uptake of InP/ZnS QDs was mainly dependent on their concentration and surface modification (Soenen et al., 2014). Moreover, the InP/ZnS QDs can be ingested by the epithelial cell line A549 (human lung carcinoma) and the neuronal cell line SH SY5Y (human neuroblastoma) (Brunetti et al., 2013). Chen et al. have investigated the *in vitro* toxicity of InP/ZnS terminated with different surface groups (−COOH, −NH_2_, and −OH, respectively) on two lung-derived cell lines, human lung cancer cell HCC-15, and Alveolar epithelial type II (AEII) cell RLE-6TN (Chen et al., 2018). They found that the intake of InP/ZnS-OH QDs in the two types of cells was relatively lower than that of the InP/ZnS-COOH QDs and InP/ZnS-NH_2_ QDs. However, the uptake mechanism of the InP/ZnS QDs toward cell membrane has been not researched so far.

Lung inhalation is a potential pathway for human exposure to quantum dots, and the lung is the first exposure target for inhaled nanoparticles. When the nanoparticles are inhaled by the human body, they are first exposed to the surface of the alveolar. The uptake of quantum dots by alveolar cells is related to the interaction between quantum dots and cell membrane (Soenen et al., 2014). The study of the effect of quantum dots on the surface behavior of the alveolar membrane is important to understand the mechanism of the uptake of quantum dots by alveolar cells and its effect on lung health.

The alveolar surface is covered with a layer of lipid secretion, called pulmonary surfactants, which is composed of phospholipids (80%), neutral lipid (8–10%), and surfactant associated proteins (10%) (Wang et al., 2018). Phosphatidylcholine (PC) comprises about 80% of total surfactant phospholipid, and dipalmitoylphosphatidylcholine (DPPC) is the most prevalent single compound (40–50% of total PC). Hydroxylated anionic phospholipids, such as phosphatidylglycerol (PG) and phosphatidylinositol (PI), constitute around 10–15% of the total lung surfactant mass (Notter et al., 2020). The pulmonary surfactant monolayer on the alveolar surface is relative to respiration, which can regulate the surface tension of the alveolar surface and promote gas exchange during respiration. The DPPC molecules play a significant role in reducing the surface tension (Veldhuizen and Haagsman, 2000). However, the spreadability of DPPC is poor, which limits the fluidity of the monolayer. The negatively charged phosphatidylglycerols-dipalmitoylphosphatidylglycerol (DPPG), another component of pulmonary surfactant, can enhance the fluidity of pulmonary surfactant and facilitate the interfacial adsorption of phospholipids and the rapid spreading of monolayer (Hallman et al., 1977). The mixed monolayer composed of DPPC and DPPG in the ratio of 4:1 (mol:mol) was widely adopted to mimic the real pulmonary surfactant monolayer (Harishchandra et al., 2009; Sachan et al., 2012; Hu et al., 2020) according to the compositional analysis of mammalian lung surfactant extracts.

In this work, the DPPC/DPPG monolayers at the air-water surface were adopted to mimic the lung surfactant monolayers and the InP/ZnS (−COOH, −NH_2_) QDs were selected as the target to study the effects of InP/ZnS QDs on the surface behavior of DPPC/DPPG monolayers by a variety of techniques, including surface pressure-mean molecular area and elastic modulus-surface pressure isotherms and surface pressure-time isotherms, as well as Brewster angle microscope and atomic force microscope observations. The effect of InP/ZnS QDs modified by different groups on surface behavior of DPPC/DPPG monolayers was explored. The results will not only give in-depth understanding of the effect of InP/ZnS QDs on surface behavior of DPPC/DPPG monolayers but also be helpful to study the harm of InP/ZnS QDs to lung health.

## Materials and Methods

### Materials

1,2-Dipalmitoyl-sn-glycero-3-phosphocholine (DPPC: purity ≥99%) and 1,2-dipalmitoyl-sn-glycero-3-phosphoglycerol sodium (DPPG: purity≥99%) were purchased from Avanti Polar Lipids (Alabaster, AL). High purity water was produced by a Milli-Q plus water purification system (18.2 MΩ/cm, Millipore, United States).

### Characterization of Quantum Dots

The aqueous InP/ZnS-COOH and InP/ZnS-NH_2_ QDs were obtained from Xi’an Qiyue Biological Technology Co., Ltd. (China). The successful functionalization of the two surface groups was analyzed by FT-IR spectra (IR Spirit, Shimadzu, Japan). The morphology images of the two InP/ZnS QDs were obtained with a transmission electron microscope (TEM) (HT7820, Hitachi, Japan) operating at an accelerating voltage of 100 kV at room temperature. The absorption spectra of InP/ZnS QDs were measured by a UV-Vis spectrophotometer (Cary 5,000, Agilent, United States). The photoluminescence emission spectra were determined by a Fluorescence spectrophotometer (FLS1000, Edinburgh, Britain) with an excitation wavelength of 378 nm.

### Methods

A Langmuir trough (KSV-Minitrough, Finland) and Wilhelmy-type tensiometer were respectively used to detect the surface behavior of the monolayer at the air-water interface. A filter paper (10 mm × 30 mm × 0.15 mm) as a pressure sensor was used in this work, and the sensor accuracy was 0.01 mN/m. A monolayer at the air-water interface of the trough subphase could be compressed or expanded symmetrically at the desired rate by using two Teflon barriers. All experiments were maintained at a controlled temperature of 35.0 ± 0.5°C which is closer to the physiological temperature of the lung surface.

#### Preparation of the Simulated Lung Surfactant Monolayer

The DPPC and DPPG with a molar ratio of 4:1 were fully dissolved in chloroform/methanol (9:1, v/v) mixture and the final concentration was about 0.5 μmol/mL. 20 μL mixture solution was deposited on the air-water interface with a Hamilton microsyringe. After 30 min of solvent evaporation, the Langmuir monolayer in equilibrium was obtained and can be used for other measurements.

#### Surface Pressure-Mean Molecular Area (π−A) Isotherms

To study the effect of InP/ZnS QDs modified by different groups on lipid monolayers, DPPC/DPPG was deposited dropwise on the surface of InP/ZnS QDs aqueous dispersion with the same concentration of 0.2 µg/ml. After 30 min, the monolayers were compressed with a constant rate of 5.25 cm^2^/min, and each measurement was repeated three times.

#### Surface Pressure-Time (π−t) Curves

The monolayers on pure water or 0.2 µg/ml InP/ZnS QDs aqueous solution were compressed to the target surface pressure (10 and 30 mN/m) with a rate of 5.25 cm^2^/min; then, the area of the monolayer was kept constant. The surface pressure-time (π−t) curves of the lipid monolayers were recorded and each measurement was repeated three times.

#### The Real-Time Observation of Monolayer’s Morphology

Brewster angle microscope (KSV NIMA, Finland) was used to detect the real-time morphology of the monolayer *in situ* visual, which is equipped with a 50 mW laser emitting p-polarized light at a wavelength of 659 nm. The image resolution is 12 μm. The maximum field of view is 3,000 μm × 4,500 μm.

#### The Microstructure of the LB Films

The Langmuir monolayers were transferred onto the new micas at 10 mN/m and 30 mN/m, forming the Langmuir-Blodgett (LB) films. The dipping rate for transfer was 7.5 mm/s. Their microstructure characterization was observed by atomic force microscope (Shimadzu, Japan) in the contacting mode using a silicon nitride pyramidal tip mounted on a 100 μm long cantilever with a force constant of 0.1 N/m.

## Results and Discussion

### Characterization of InP/ZnS Quantum Dots

**FIGURE 1:**
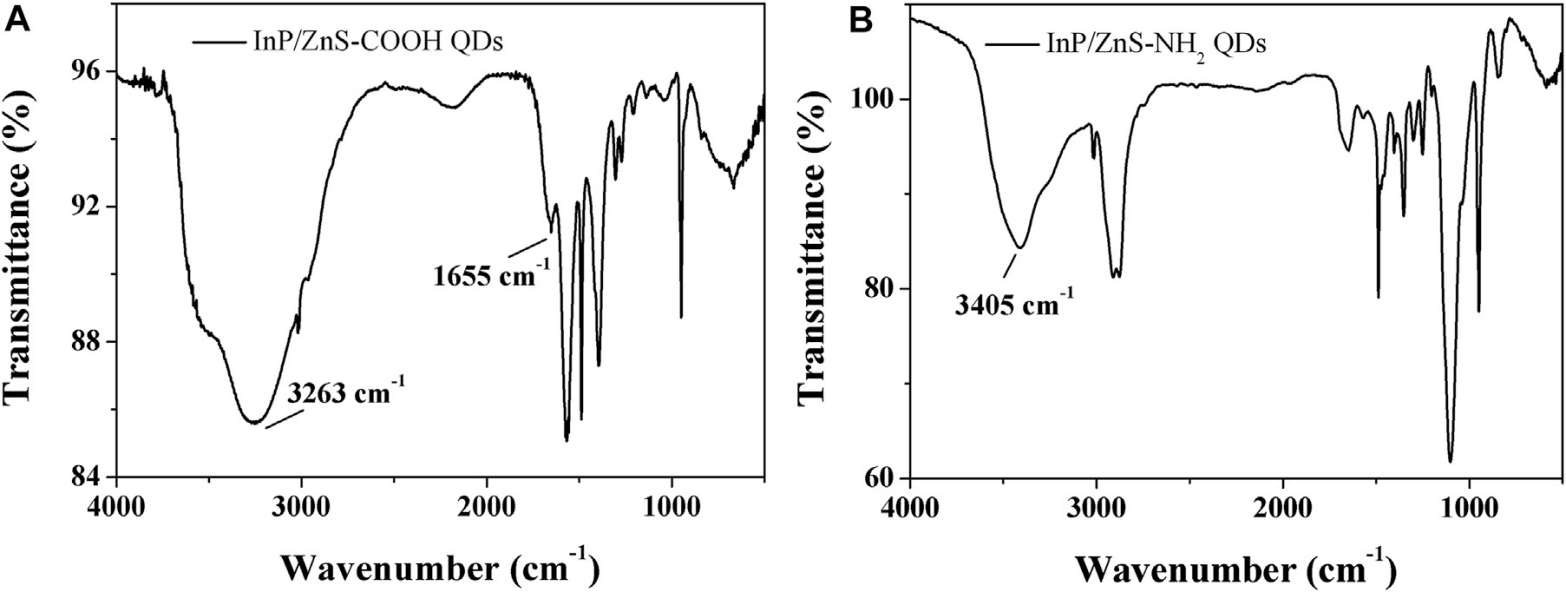
Infrared spectrum of InP/ZnS QDs coated with carboxyl **(A)** and amino **(B)** groups.

**FIGURE 2:**
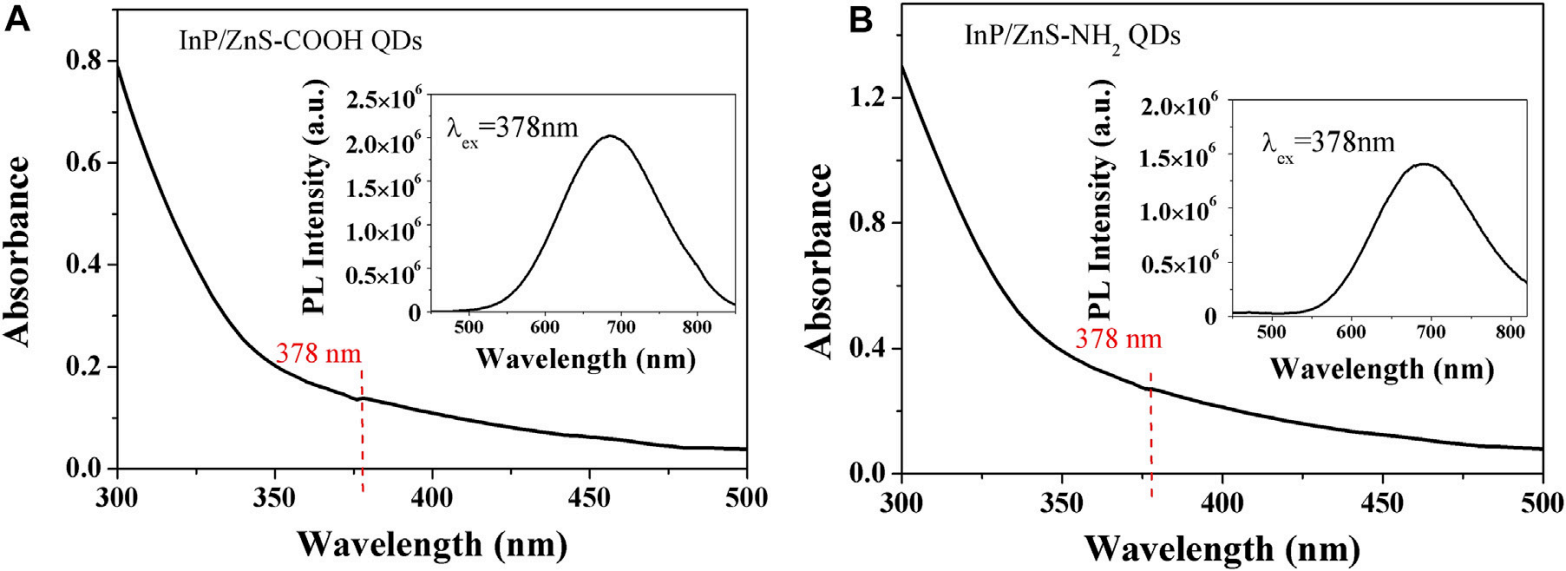
Absorption spectra and photoluminescence spectra of InP/ZnS QDs coated with carboxyl **(A)** and amino **(B)** groups.

**FIGURE 3:**
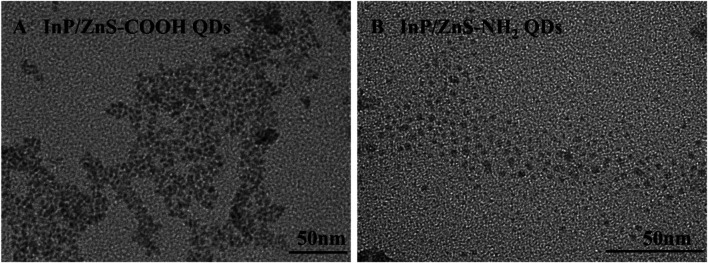
The TEM images of InP/ZnS QDs coated with carboxyl **(A)** and amino **(B)** groups.

The infrared spectra analysis of the InP/ZnS QDs modified with carboxyl and amino groups were performed, indicating successful functionalization of the two surface groups (Figure 1). The appearance of the band at 1,655 cm^−1^ corresponded to the stretching vibration of C=O for the coated QDs. The strong band at 3,263 cm^−1^ was assigned to the OH stretching vibration of COOH (Figure A). The characteristic band at 3,405 cm^−1^ corresponded to the stretching vibration of N-H present in NH_2_ (Figure B). From Figure 2, the two QDs exhibited consistent absorption spectra with the same absorption peak around 378 nm. Under exciting by 378 nm light source, the two QDs all exhibited relatively symmetrical photoluminescence spectra with the emission peak around 690 nm. The TEM images of the two QDs are shown in Figure 3. It demonstrated a relatively monodispersed size distribution with an average size of ～5 nm.

### Surface Pressure-Mean Molecular Area (π−A) Isotherms and the Elastic Modulus-Surface Pressure ($C_{s}^{-1}-\pi$) Isotherms

**FIGURE 4:**
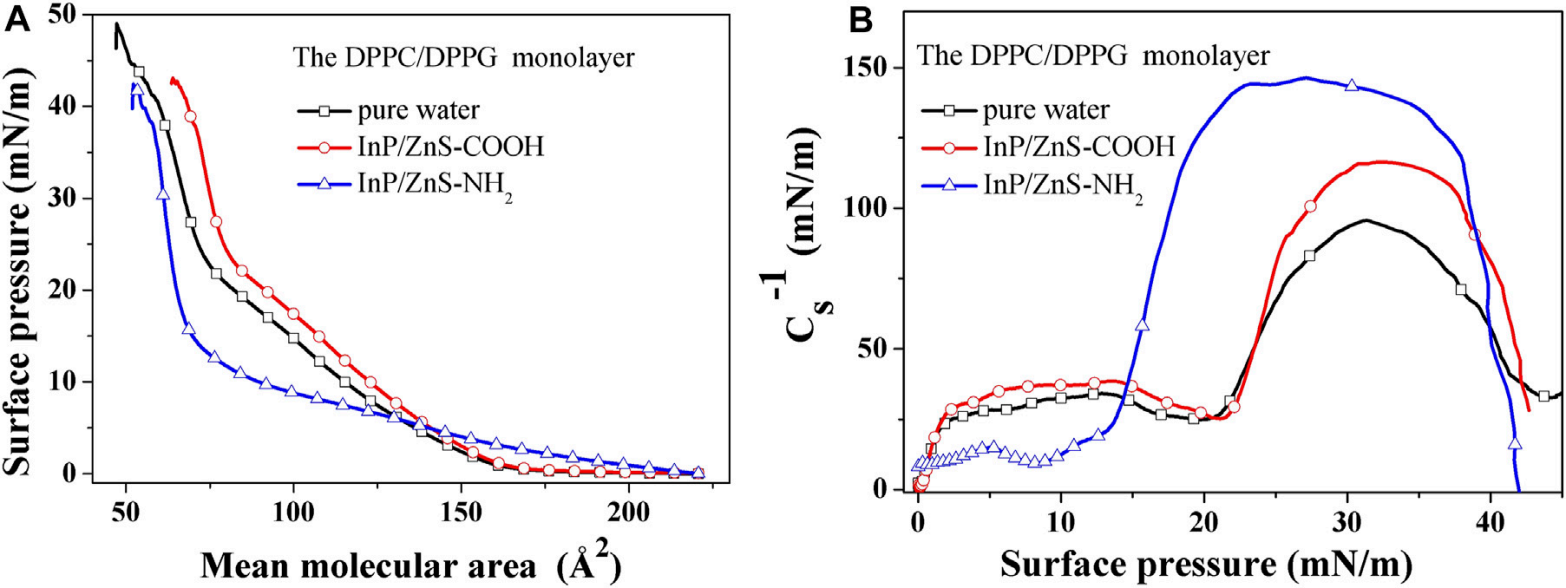
Surface pressure-mean molecular area (π−A) isotherms **(A)** and the elastic modulus-surface pressure ($C_{s}^{-1}-\pi$) isotherms **(B)** of DPPC/DPPG (4:1, mol/mol) monolayers on subphase of pure water and with 0.2 μg/mL InP/ZnS (−COOH, −NH_2_) QDs at 35.0 ± 0.5°C.

The π−A isotherm can be used to suggest the surface behavior information of the monolayer. As shown in Figure 4A, for the mixed DPPC/DPPG (4:1, mol/mol) monolayer spread on the pure water, it can be seen that the mixed monolayer did not show obvious plateaus that corresponded to the two-phase coexistence region (Song et al., 2007; Nakahara et al., 2008; Harishchandra et al., 2010; Wang et al., 2018). The surface pressure increased slightly with the decrease in the mean molecular area when the mean molecular area was larger ($160{Å}^{2}>\text{A}>74{Å}^{2}$), which corresponded to the liquid expanded (LE) phase. As the monolayer was compressed further ($74{Å}^{2}>\text{A}>41{Å}^{2}$), the surface pressure increased significantly, which corresponded to the liquid condensed (LC) phase. An obvious inflection point appeared at about 41 mN/m before the collapse of the mixed monolayer. The trend of π−A isotherm for DPPC/DPPG mixed monolayer on the pure water was similar to the literature (Hu et al., 2020), but the corresponding mean molecular area region was different. This was because our experimental temperature was 35°C, while the experimental temperature in the literature was 20°C. When the InP/ZnS-COOH quantum dots nanoparticles were dispersed in water, the shape of the π−A isotherm for DPPC/DPPG mixed monolayer was similar to that on the surface of pure water. The difference was that the InP/ZnS-COOH QDs caused the mean molecular area to increase at the same surface pressure. However, the DPPC/DPPG mixed monolayer was compressed on the surface of water in the presence of InP/ZnS-NH_2_ QDs; the shape of the π−A isotherm changed significantly. The isotherm rose at the mean molecular area of 210 ${Å}^{2}$, which was greater than that compressed on the pure water in the absence and presence of InP/ZnS QDs. The surface pressure increased slightly with the decrease in the mean molecular area from 220 ${Å}^{2}$ to 68 ${Å}^{2}$ and the surface pressure increased significantly with the decrease in the mean molecular area from 68 ${Å}^{2}$ to 56 ${Å}^{2}$.

According to the data of π−A curves, the elastic modulus of the monolayer can be calculated by the formula (Davies and Rideal, 1963; Panda et al., 2005):

$$C_{s}^{-1}=-A{\left(\frac{d\pi }{dA}\right)}_{T}$$ (1)

where $C_{s}^{-1}$ is the elastic modulus of the monolayer, *s* is the cross-sectional area of the monolayer, *A* is the mean molecular area, and *π* is the surface pressure of the monolayer. A greater elastic modulus suggests a less compressible monolayer, corresponding to the more condensed monolayer (Gopal et al., 2006). The minimum of $C_{s}^{-1}$ indicates a significant phase transition in the monolayer (Gzyl-Malcher et al., 2012).

The elastic modulus-surface pressure ($C_{s}^{-1}-\pi$) isotherms of DPPC/DPPG (4:1, mol/mol) monolayers have be shown in Figure 4B. In the presence of InP/ZnS-COOH QDs, the value of $C_{s}^{-1}$ reached the minimum at the surface pressure of 21.1 mN/m, which was closed to the surface pressure (20.1 mN/m) corresponding to the minimum of $C_{s}^{-1}$ in the absence of InP/ZnS QDs. Different significantly, in the presence of InP/ZnS-NH_2_ QDs, the surface pressure corresponding to the minimum value of $C_{s}^{-1}$ reduced to 7.9 mN/m. This suggested that the InP/ZnS QDs modified by the −COOH group had almost no effect on the phase transition point of the DPPC/DPPG mixed monolayer. At the region of surface pressure from 1.4 mN/m to 20.3 mN/m and from 24 mN/m to 41 mN/m, the $C_{s}^{-1}$ values in the presence of InP/ZnS-COOH QDs were greater than those in the absence of InP/ZnS QDs. However, the $C_{s}^{-1}$ values in the presence of InP/ZnS-NH_2_ QDs were smaller at the region of surface pressure from 0mN/m to 14.4mN/m but were greater obviously at the region of surface pressure from 14.4 mN/m to 39.8 mN/m than that in the absence of InP/ZnS QDs. It has been suggested that the InP/ZnS QDs modified by the −COOH group caused the DPPC/DPPG mixed monolayer to be more condensed at the above-mentioned range of surface pressures, which was similar to that in the presence of the InP/ZnS QDs modified by the −NH_2_ group at the higher surface pressures. Differently, the InP/ZnS QDs modified by the −NH_2_ group induced the mixed monolayer less condensed at the lower surface pressures. The effect of quantum dots on the elastic modulus of DPPC/DPPG mixed monolayer was not only related to the modified group of quantum dots but also related to the surface pressure.

### Surface Pressure-Time (π−t) Curves

**FIGURE 5:**
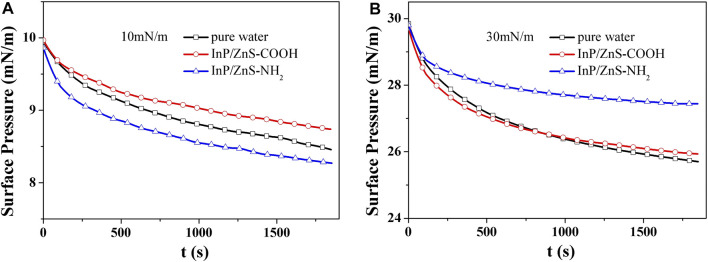
Surface pressure-time (π−t) curves of DPPC/DPPG (4:1, mol/mol) monolayers at the initial surface pressure of 10 mN/m **(A)** and 30 mN/m **(B)** on subphase of pure water and with 0.2 μg/mL InP/ZnS (−COOH, −NH_2_) QDs at 35.0 ± 0.5°C.

Compressing the DPPC/DPPG mixed monolayer to the specific surface pressure (10 mN/m and 30 mN/m) on the surface of water without or with the InP/ZnS QDs and keeping the area of the monolayer being constant, the changes of surface pressure with time were recorded for 30 min, which are shown in Figure 5. The surface pressure decreased or increased until the equilibrium value (${\text{π}}_{e}$) was reached (Wang et al., 2020). The initial surface pressure was ${\text{π}}_{i}$ and the final change of surface pressure Δπ was calculated by the formula $\Delta \pi =\left|\pi_{e}-\pi_{i}\right|$. The smaller value of Δπ indicated that the monolayer is more stable.

At the surface pressure of 10 mN/m (Table 1), the presence of InP/ZnS-COOH QDs caused the Δπ value to decrease, which suggested the InP/ZnS-COOH QDs enhanced the stability of DPPC/DPPG mixed monolayer. On the contrary, InP/ZnS-NH_2_ QDs induced the Δπ value to increase, which indicated that InP/ZnS-NH_2_ QDs weaken the stability of DPPC/DPPG mixed monolayer. Different significantly, at the surface pressure of 30 mN/m, the InP/ZnS-NH_2_ QDs caused the Δπ value to decrease obviously, which suggested that InP/ZnS-NH_2_ QDs can enhance the stability of DPPC/DPPG mixed monolayer. When in the presence of InP/ZnS-COOH QDs, the Δπ values increased in 14 min but decreased after 15 min, which indicated that the effect of InP/ZnS-COOH QDs on the stability of DPPC/DPPG mixed monolayer may be relative to the time exposed to the quantum dots environment. But the influence of InP/ZnS-COOH QDs was less intense than that of InP/ZnS-NH_2_ QDs.

**TABLE 1:** Relevant characteristic parameters of the DPPC/DPPG mixed monolayer spread on the surface of different subphases.

Surface pressure (mN/m)	Subphase	Mean molecular area a (${Å}^{2}$)	Elastic modulus $C_{s}^{-1}$ (mN/m)	Change of surface pressure Δπ (mN/m)
10	Pure water	115.36 ± 0.05	32.41 ± 0.04	1.61 ± 0.04
	InP/ZnS-COOH	122.72 ± 0.03	37.23 ± 0.05	1.32 ± 0.04
	InP/ZnS-NH_2_	89.21 ± 0.05	12.72 ± 0.04	1.81 ± 0.06
30	Pure water	67.33 ± 0.04	93.45 ± 0.03	4.33 ± 0.05
	InP/ZnS-COOH	75.15 ± 0.05	113.83 ± 0.06	4.15 ± 0.07
	InP/ZnS-NH_2_	61.13 ± 0.07	143.64 ± 0.07	2.61 ± 0.04

### Real-Time Observation of Monolayer’s Morphology

**FIGURE 6:**
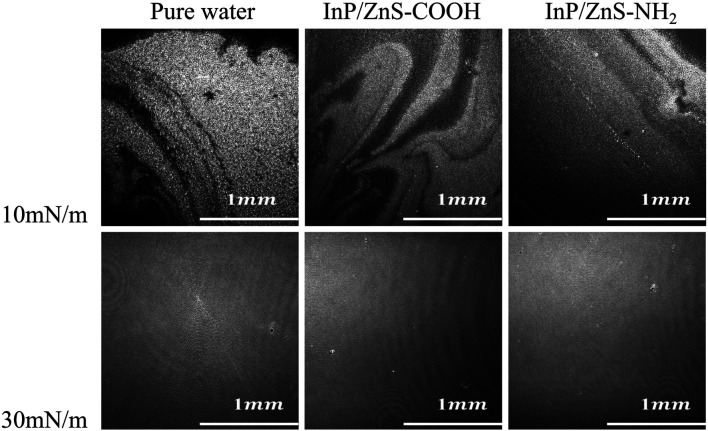
The BAM images (2000 μm × 2000 μm) of the mixed DPPC/DPPG (4:1, mol ratio) monolayers spread on the pure water and in the presence of InP/ZnS QDs modified by −COOH or −NH_2_ group at 10 mN/m and 30 mN/m.

Further characterization of the effect of InP/ZnS quantum dots on the morphology of the DPPC/DPPG mixed monolayers was performed in real time by using BAM. Figure 6 shows the BAM images for the mixed monolayers at 10 mN/m and 30 mN/m in the absence and presence of InP/ZnS QDs modified by −COOH or −NH_2_ group. In the BAM images, the bright areas are the regions of monolayer and the dark areas are the surface of subphase water. At 10 mN/m, dark areas were observed in the regions of DPPC/DPPG mixed monolayer spread on the surface of pure water, which corresponded to the LE phase. The larger the dark areas, the less compact the monolayers. When the density of the mixed monolayer regions is small, the mean molecular area is small. In the presence of InP/ZnS QDs modified by the −COOH group, the dark areas were larger, which was consistent with the larger molecular area (122.72 ${Å}^{2}$ seen in Table 1). And in the presence of InP/ZnS QDs modified with the −NH_2_ group, the dark areas were smaller, which was consistent with the smaller molecular area (89.21 ${Å}^{2}$). At 30 mN/m, the morphology of the DPPC/DPPG mixed monolayer looked very tight and flat, which corresponded to the LC phase.

### Microstructural Information of the LB Films

**FIGURE 7:**
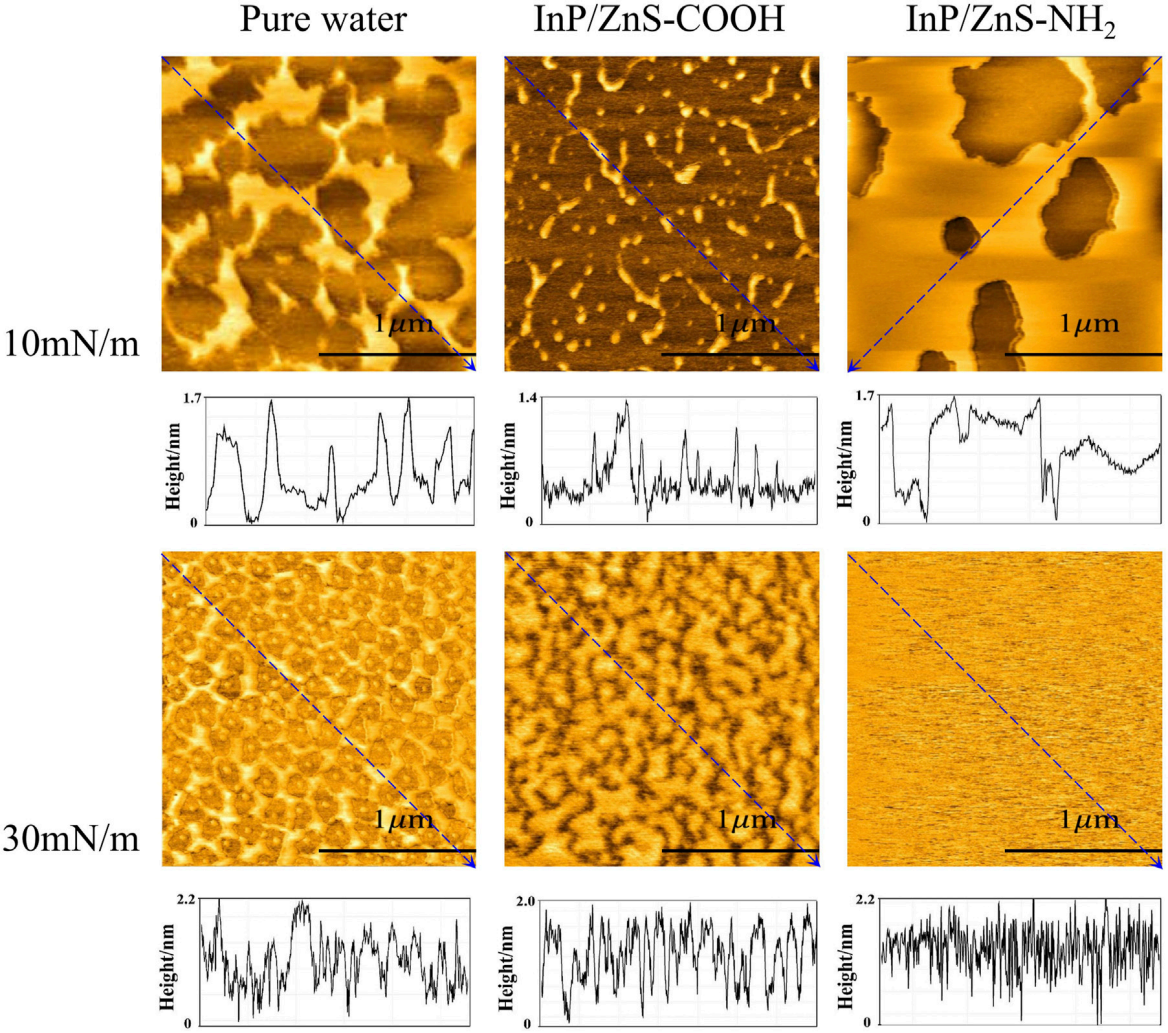
The AFM images (2 μm × 2 μm) and height analysis of the DPPC/DPPG (4:1, mol ratio) mixed monolayer films transferred onto the micas, which were spread on the pure water and in the presence of InP/ZnS QDs modified with −COOH or −NH_2_ group at 10 mN/m and 30 mN/m.

**FIGURE 8:**
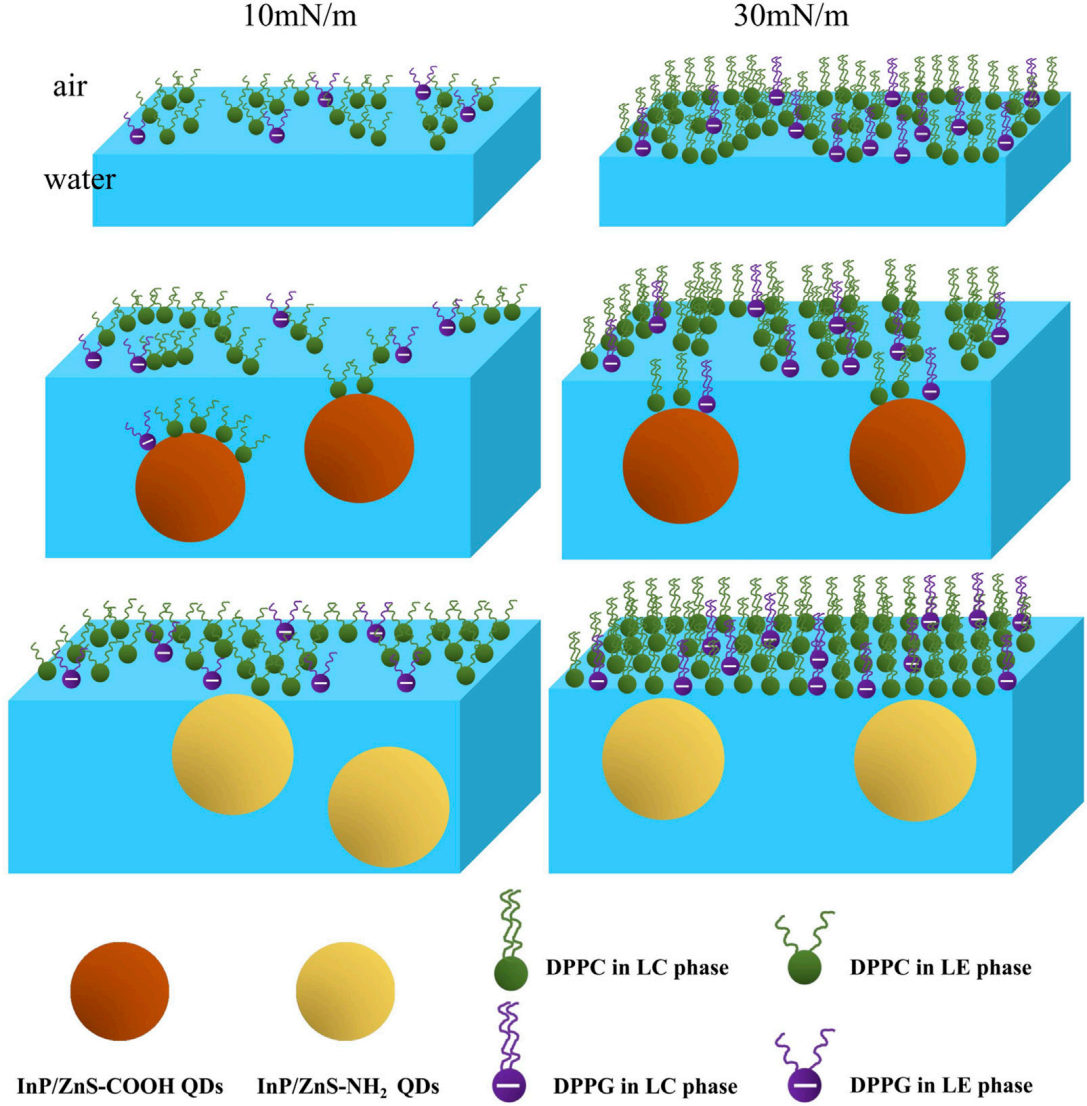
Schematic diagram of the DPPC/DPPG mixed monolayer at 10 mN/m and 30 mN/m in the absence and presence of InP/ZnS-COOH and InP/ZnS−NH_2_ QDs.

The microstructure information of mixed monolayers was not available by the above BAM images due to the image resolution of 12 μm. So, we transferred the monolayers onto the surface of micas to be films. The microstructure of the monolayer films was observed by an atomic force microscope (Figure 7), and the root mean square roughness (RMS-Rq) and roughness average (Ra) of AFM images were shown in Table 2. At 10 mN/m, when the monolayer was compressed on the pure water, the shape of the microregions in the film looked like “islands” and the island regions were not very tightly distributed. When the monolayer was spread on the water in the presence of InP/ZnS-COOH QDs, the shape of the microregions in the film were shaped like “narrow discontinuous chains” and the “chains” regions were far apart from each other. It can be suggested that the InP/ZnS-COOH QDs decreased the density of the films, which was in agreement with the larger mean molecular area (75.15 ${Å}^{2}$ seen in Table 1) than that in the case of pure water (67.33 ${Å}^{2}$). The InP/ZnS-COOH QDs reduced the height of the lipid monolayer, as seen in the height curves of the AFM image. This may be due to the interaction between the nanoparticles and the head groups of phospholipids, pulling the phospholipid molecules from the interface into the subphase solution (shown in the schematic diagram of Figure 8). In the presence of InP/ZnS-NH_2_ QDs, the microregions in the film were larger and connected in “sheets” with some holes, which was consistent with the smaller mean molecular area (61.13 ${Å}^{2}$) than that in the case of pure water. The effect of InP/ZnS-NH_2_ QDs on the height of lipid monolayer was not obvious, which may be because the interaction force between the nanoparticles and the head groups of phospholipids at the interface was not strong enough, and the phospholipid molecules may not be pulled from the interface into the subphase solution. But the InP/ZnS-NH_2_ QDs can enhance the distribution density of phospholipid molecules at the interface (shown in the schematic diagram of Figure 8).

**TABLE 2:** The root mean square roughness (RMS-R_q_) and roughness average (R_a_) in AFM images.

Surface pressure (mN/m)	Subphase	RMS-R_q_ (nm)	R_a_ (nm)
10	Pure water	0.788 ± 0.012	0.619 ± 0.010
	InP/ZnS-COOH	0.317 ± 0.010	0.243 ± 0.009
	InP/ZnS-NH_2_	0.251 ± 0.013	0.162 ± 0.011
30	Pure water	0.271 ± 0.011	0.204 ± 0.012
	InP/ZnS-COOH	0.243 ± 0.012	0.197 ± 0.013
	InP/ZnS-NH_2_	0.432 ± 0.011	0.327 ± 0.014

At 30 mN/m, the structure of DPPC/DPPG mixed monolayer films was more orderly and compact than that at 10 mN/m. When the monolayer was spread on pure water, the microregions of the film formed a “network.” The shape of its mesh was irregular and there was a small nearly circular “island” region inside each mesh. The height of the lipid monolayer was larger than that at 10 mN/m, which is because the hydrophobic tail chain of phospholipid molecules was straightened at 30 mN/m. In the presence of InP/ZnS-COOH QDs, the shape of microregions was irregular and looked like some “islands” that were close to each other. In the presence of InP/ZnS-NH_2_ QDs, the films were very dense and uniform with some irregular dark pores faintly observed. The effect of these two QDs on the height of the lipid monolayer was similar to that at10 mN/m.

According to the mean molecular areas and AFM analysis of the DPPC/DPPG mixed monolayer, the schematic diagram at the air-water interface, corresponding to the morphology of the monolayer films in AFM images, is in Figure 8, which may help us to understand the effect of InP/ZnS QDs on the distribution of DPPC/DPPG mixed monolayer. In conclusion, it can be suggested that the effect of InP/ZnS QDs on the microstructure of DPPC/DPPG mixed monolayer is different due to the different modified groups and the different surface pressure (or different phase state of the monolayer).

## Conclusion

In conclusion, the effects of −COOH and −NH_2_ groups modified InP/ZnS QDs on the surface behavior and morphology of the DPPC/DPPG (4:1, mol/mol) mixed monolayer at the air-water interface has been systematically studied. The Langmuir experiments suggested the InP/ZnS QDs with different groups had different effects on the mean molecular areas, elastic modulus, phase transition point, and stability of the DPPC/DPPG mixed monolayer. The results from BAM and AFM images indicated the different influences of InP/ZmS QDs on the morphology of mixed monolayer due to QDs’ groups and monolayers’ phase states. The effect of InP/ZnS-NH_2_ QDs on the π−A isotherms of the mixed monolayer was more significant than that of InP/ZnS-COOH QDs. The InP/ZnS QDs modified by the −COOH group caused the DPPC/DPPG mixed monolayer to be more condensed, but the InP/ZnS QDs modified by the −NH_2_ group induced the mixed monolayer less condensed at the lower surface pressures. In the LE phase, the stability of DPPC/DPPG mixed monolayer was enhanced by InP/ZnS-COOH QDs but was weaken by InP/ZnS-NH_2_ QDs. Differently in the LC phase, the stability of DPPC/DPPG mixed monolayer was all enhanced by the InP/ZnS QDs, which did not depend on the modified groups. But the effect was greater in the presence of −NH_2_ groups. The InP/ZnS QDs can affect the morphology of the DPPC/DPPG mixed monolayer, which was different depending on the modified groups and the different phase state of the monolayer. The results will be helpful to understand the effect of InP/ZnS QDs nanoparticles modified by −COOH groups and −NH_2_ groups on the surface of the alveolar membrane. Furthermore, it also provides a new research idea at a membrane level for studying the impact of QDs on the lungs.

The results of this work help us to understand the effects of fluorescent quantum dots on pulmonary surfactant monolayer on the alveolar surface during respiration. If the fluorescent quantum dots are inhaled into the lungs, especially when they are used in living lung imaging or detection, the expansion behavior and morphology of pulmonary surfactant monolayer may be affected, which may damage the health of the lung. This work has shown that the effects of InP/ZnS QDs modified -carboxyl or -amino on the pulmonary surfactant monolayer are significantly different. The relationship between these different effects and the functional changes of pulmonary surfactant monolayer on the alveolar surface is not clear now, which needs to be further explored. In addition, the adsorption of quantum dots on the pulmonary surfactant monolayer is also worthy of careful study to indicate the adsorption behavior and mechanism of quantum dots on the alveolar surface. Our work in the future will involve the following two points: 1) the adsorption kinetics of quantum dots on the pulmonary surfactant monolayer; 2) the effect of quantum dots on phase behavior and morphology of the pulmonary surfactant monolayer at different temperatures (35～45°C) and pH conditions. More detailed information on the interaction of quantum dots with a pulmonary surfactant monolayer on the alveolar surface during respiration will be obtained, helping us understand the more details close to the real situation.

## Data Availability

The original contributions presented in the study are included in the article/supplementary material; further inquiries can be directed to the corresponding authors.
